# Blood pressure levels and cardiovascular risk according to age in patients with diabetes mellitus: a nationwide population-based cohort study

**DOI:** 10.1186/s12933-020-01156-8

**Published:** 2020-10-19

**Authors:** Hack-Lyoung Kim, Hyue Mee Kim, Chang Hee Kwon, Jeong-Hun Shin, Mi-Hyang Jung, Chan Joo Lee, Dae-Hee Kim, Woo-Hyeun Kim, Si-Hyuck Kang, Ju-Hee Lee, In Jeong Cho, Iksung Cho, Jun Hyeok Lee, Dae Ryong Kang, Hae-Young Lee, Wook-Jin Chung, Sang-Hyun Ihm, Kwang Il Kim, Eun Joo Cho, Il-Suk Sohn, Hyeon-Chang Kim, Jinho Shin, Ju Han Kim, Sung Kee Ryu, Seok-Min Kang, Wook Bum Pyun, Myeong-Chan Cho, Sungha Park, Ki-Chul Sung

**Affiliations:** 1 Division of Cardiology, Department of Internal Medicine, Boramae Medical Center, Seoul National University College of Medicine, Seoul, Republic of Korea; 2 Division of Cardiology, Department of Internal Medicine, Chung-Ang University Hospital, Chung-Ang University, Seoul, Republic of Korea; 3 Department of Internal Medicine, Konkuk University Medical Center, Konkuk University School of Medicine, Seoul, Republic of Korea; 4 Division of Cardiology, Department of Internal Medicine, Hanyang University College of Medicine, Seoul, Republic of Korea; 5 Cardiovascular Center, Dongtan Sacred Heart Hospital, Hallym University College of Medicine, Hwaseong, Republic of Korea; 6 Division of Cardiology, Severance Cardiovascular Hospital and Cardiovascular Research Institute, Yonsei University College of Medicine, 50-1, Yonsei-ro, Seodaemun-gu, Seoul, 03722 Republic of Korea; 7 Division of Cardiology, Asan Medical Center, University of Ulsan College of Medicine, Seoul, Republic of Korea; 8 Cardiovascular Center, Korea University Guro Hospital, Seoul, Republic of Korea; 9 Department of Internal Medicine, Seoul National University Bundang Hospital, Seoul National University College of Medicine, Seongnam, Republic of Korea; 10 Division of Cardiology, Department of Internal Medicine, Chungbuk National University Hospital, Chungbuk National University College of Medicine, Cheongju, Republic of Korea; 11 Division of Cardiology, Department of Internal Medicine, Ewha Womans University Medical Center, Seoul, Republic of Korea; 12 Center of Biomedical Data Science, Wonju College of Medicine, Yonsei University, Wonju, Republic of Korea; 13 Division of Cardiology, Department of Internal Medicine, Seoul National University Hospital, Seoul, Republic of Korea; 14 Division of Cardiology, Department of Internal Medicine, Gil Hospital, Gachon University, Incheon, Republic of Korea; 15 Division of Cardiology, Department of Internal Medicine, Bucheon St. Mary’s Hospital, The Catholic University of Korea, Bucheon, Republic of Korea; 16 Division of Cardiology, Department of Internal Medicine, Yeouido St. Mary’s Hospital, The Catholic University of Korea, Seoul, Republic of Korea; 17 Division of Cardiology, Department of Internal Medicine, KyungHee University at Gangdong, Seoul, Republic of Korea; 18 Division of Cardiology, Department of Internal Medicine, Chonnam National University Hospital, Gwangju, Republic of Korea; 19 Division of Cardiology, Department of Internal Medicine, Eulji Medical School of Medicine, Seoul, Republic of Korea; 20 Division of Cardiology, Department of Internal Medicine, Kangbuk Samsung Hospital, Sungkyunkwan University School of Medicine, 29 Saemunan-ro, Jongno-gu, Seoul, 03181 Republic of Korea

**Keywords:** Age, Cardiovascular risk, Diabetes mellitus, Hypertension, Target blood pressure

## Abstract

**Background:**

Little is known about age-specific target blood pressure (BP) in hypertensive patients with diabetes mellitus (DM). The aim of this study was to determine the BP level at the lowest cardiovascular risk of hypertensive patients with DM according to age.

**Methods:**

Using the Korean National Health Insurance Service database, we analyzed patients without cardiovascular disease diagnosed with both hypertension and DM from January 2002 to December 2011. Primary end-point was composite cardiovascular events including cardiovascular death, myocardial infarction and stroke.

**Results:**

Of 241,148 study patients, 35,396 had cardiovascular events during a median follow-up period of 10 years. At the age of < 70 years, the risk of cardiovascular events was lower in patients with BP < 120/70 mmHg than in those with BP 130–139/80–89 mmHg. At the age of ≥ 70, however, there were no significant differences in the risk of cardiovascular events between patients with BP 130–139/80–89 mmHg and BP < 120/70 mmHg. The risk of cardiovascular events was similar between patients with BP 130–139/80–89 mmHg and BP 120–129/70–79 mmHg, and it was significantly higher in those with BP ≥ 140/90 mmHg than in those with BP 130–139/80–89 mmHg at all ages.

**Conclusions:**

In a cohort of hypertensive patients who had DM but no history of cardiovascular disease, lower BP was associated with lower risk of cardiovascular events especially at the age of < 70. However, low BP < 130–139/80–89 mmHg was not associated with decreased cardiovascular risk, it may be better to keep the BP of 130–139/80–89 mmHg at the age of ≥ 70.

## Background

Hypertension and diabetes mellitus (DM), 2 major cardiovascular risk factors, have emerged as major medical and public health issues globally. There has been a continued growth in the prevalence of hypertension [1] and DM [2], and both conditions are associated with increased risk of cardiovascular morbidity and mortality [3–6]. Hypertension affects approximately 70% of patients with DM, which is twice as common as those without DM [7]. Importantly, the coexistence of hypertension and DM substantially increases in the risk of cardiovascular disease (CVD), and chronic kidney disease [8, 9]. Two thirds of diabetic patients die from CVD, in which hypertension is the main cause of CVD [10]. Therefore, it is very important to control hypertension in patients with DM in order to reduce their cardiovascular risk and to improve prognosis.

Because blood pressure (BP) rises with age, hypertension is one of the main medical problem with high prevalence in the elderly [11]. Even in elderly people, the beneficial effect of BP control on the reduction in the risk of cardiovascular events has been suggested [12, 13], and BP control should not be neglected. However, elderly subjects are often frail, have many comorbidities, and are more vulnerable to the side effects of intensive BP control [14]. In clinical practice, many physicians are worried about the side effects or complications of intensive BP lowering in elderly patients. Therefore, age must be considered when setting target BP. However, there is limited data regarding age-specific target BP in patients with DM. The 2017 American College of Cardiology/American Heart Association (ACC/AHA) guidelines suggested a target BP of 130/80 mmHg for diabetic patients at all ages [15]. Otherwise, in patients with DM, the European guidelines recommended a target BP of 130/70–79 mmHg and 130–140/70–79 mmHg for subjects aged < 65 years and ≥ 65 years, respectively [16]. There is still insufficient evidence as to whether BP needs to be lowered intensively in patients with DM and whether the target BP should differ according to age. Thus, the aim of this study was to determine the BP level at the lowest cardiovascular risk of hypertensive patients with DM according to age.

## Methods

### Data sources

This study used a database provided by the National Health Insurance Services-Health Screening (NIHS-HEALS) cohort in Korea. NHIS is a single insurance provider in Korea and covers 97.2% of the Korean population; enrollees aged 40 years or older are entitled to a general health screening program every 2 years. A database includes data regarding sociodemographics, use of inpatient and outpatient services, diagnoses, prescriptions, death, and health screening examination data (e.g., health questionnaires and laboratory tests). The cohort details have been previously described [17]. The study was approved by the Institutional Review Board of Kangbuk Samsung Hospital (# KBSMC 2019-01-018). The anonymized dataset was provided to the researchers from the NHIS and informed consent was waived.

### Study population and patient involvement

**Fig. 1:**
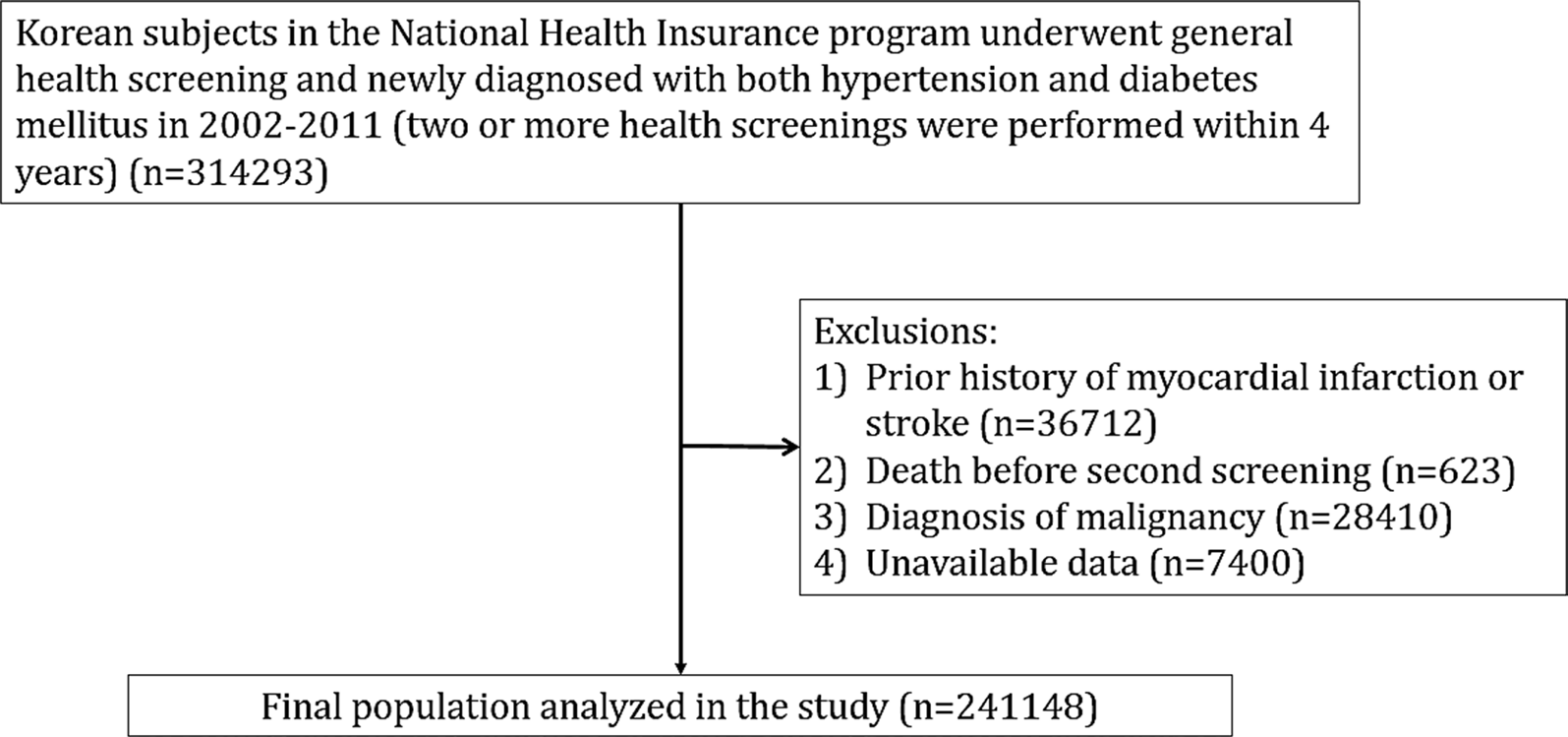
Study flowchart showing patient enrollment

A total of 314,293 subjects who were diagnosed with both hypertension and DM from January 2002 to December 2011 were extracted from the NIHS-HEALS cohort. Subjects were considered as having hypertension if: (1) hypertension was diagnosed before health screening examination, or (2) anti-hypertensive medications were prescribed before. Having DM was defined if: (1) DM was diagnosed before health screening examination, (2) hypoglycemic agents were prescribed before, or (3) fasting glucose ≥ 126 mg/dL. Among them, patients with the following were excluded: prior history of myocardial infarction or stroke (n = 36,712), death before second screening (n = 623), diagnosis of malignancy (n = 28,410), and unavailable data (n = 7400). Therefore, a total of 241,148 patients were finally analyzed. Flow chart for study enrollment is shown in Fig. 1. Individuals were followed up until the development of death, and the first occurrence of cardiovascular events, or the end of the study (December 2017). This research was done without patient involvement.

### BP data

The data on BP records were extracted from the NIHS-HEALS cohort. Considering BP variability, 2 BP records within 4 years were averaged. BP measured using a sphygmomanometer or an oscillometric device. BP measurements were recommended twice at 2 min intervals after 5 min of stabilization.

### Cardiovascular events

The collection of information on the occurrence of cardiovascular events began the day after second check-up, and patients with cardiovascular events between 2 check-ups were excluded from the study. Baseline clinical data used in this study was obtained from the second check-up. The primary study endpoint was major cardiovascular events including cardiovascular death, myocardial infarction and stroke. A diagnosis of myocardial infarction was made based on discharge diagnosis after a hospitalization (ICD-10 codes: I21-23). A diagnosis of stroke was made based on discharge diagnosis (ICD-10 codes: I60-69) in patients who had been hospitalized and undergone brain imaging [18]. The cause and date of death were confirmed by the records from the National Statistical Office of Korea. The secondary study endpoint was each clinical event.

### Statistical analysis

Continuous variables are expressed as mean (standard deviation) and categorical variables as percentages. The mean values of continuous variables were compared using analysis of variance, and the frequencies of categorical variable were compared using chi-square test among BP category groups. The incidence of endpoints was calculated using the total number of outcomes during the follow-up period divided by 100,000 person-years. Multivariable analysis was performed using the Cox proportional hazard model to evaluate the relationship of BP with the cardiovascular events and mortality. Hazard ration (HR) and 95% confidence interval (CI) were calculated and adjusted for age, income level, history of smoking, physical activity, alcohol consumption, body mass index, fasting glucose, total cholesterol, and use of aspirin or statin. Subgroup analyses were performed by dividing the patients into those aged < 50 years, 50–59 years, 60–69 years, and ≥ 70 years to determine the appropriate target BP according to age. Restricted cubic splines were fitted to evaluate the non-linear relationship between BP and outcomes. *P* value < 0.05 was considered statistically significant. Statistical analyses were performed using SAS Statistical Software (version 9.4, SAS Institute, Cary, North Carolina, USA) and R Statistical Software (version 3.5.2, R Foundation for Statistical Computing, Vienna, Austria).

## Results

### Baseline characteristics of the study patients according to BP categories

The baseline characteristics of the study subjects according to BP categories are shown in Table 1. Compared to the lower BP group, the higher BP group tended to be older and male, had higher BMI, consumed more alcohol, had low household income, had higher level of fasting glucose and total cholesterol, and more frequently used aspirin or antihypertensive medications, and less frequently used statin.

**Table 1:** Baseline characteristics of study subjects according to SBP/DBP categories

Characteristic	< 120/ < 70 mmHg		120–129/70–79 mmHg		130–139/80–89 mmHg		140–149/90–99 mmHg		≥ 150/ ≥ 100 mmHg		*P* value	Total	
N	10,077	4.18%	59,116	24.51%	99,595	41.30%	49,915	20.70%	22,445	9.31%		241,148	100%
Age, median (IQR), years	56 (48–64)		57 (49–64)		57(49–64)		59 (50–66)		62 (53–68)		< 0.001	58 (50–66)	
Sex											< 0.001		
Men	3604	35.76%	27,612	46.71%	52,940	53.16%	26,277	52.64%	11,374	50.67%		121,807	50.51%
Women	6473	64.24%	31,504	53.29%	46,655	46.84%	23,638	47.36%	11,071	49.33%		119,341	49.49%
Body mass index, mean (SD), kg/m^2^	23.31 (3.06)		24.49 (3.15)		25.12 (3.18)		25.35 (3.29)		25.33 (3.51)		< 0.001	24.96 (3.26)	
< 18.5	479	4.75%	1158	1.96%	1224	1.23%	560	1.12%	353	1.57%		3774	1.57%
18.5–22.9	4269	42.36%	17,432	29.49%	22,792	22.88%	10,728	21.49%	5146	22.93%		60,367	25.03%
23.0–24.9	2507	24.88%	15,863	26.83%	25,771	25.88%	12,251	24.54%	5330	23.75%		61,722	25.60%
≥ 25.0	2822	28.00%	24,663	41.72%	49,808	50.01%	26,376	52.84%	11,616	51.75%		115,285	47.81%
BP, mean (SD), mmHg													
Systolic BP	110.03 (6.14)		121.59 (5.43)		132.06 (4.91)		142.87 (4.01)		156.98 (7.87)		< 0.001	133.13	
Diastolic BP	65.68 (3.19)		74.72 (3.35)		81.81 (4.21)		86.82 (5.63)		91.92 (7.77)		< 0.001	81.38	
Smoking											< 0.001		
Never	7320	72.64%	39,755	67.25%	65,049	65.31%	33,616	67.35%	15,748	70.16%		161,488	66.97%
Past	1211	12.02%	9563	16.18%	18,138	18.21%	8629	17.29%	3271	14.57%		40,812	16.92%
Current	1546	15.34%	9798	16.57%	16,408	16.47%	7670	15.37%	3426	15.26%		38,848	16.11%
Physical activity, times/week											< 0.001		
0	5461	54.19%	31,021	52.47%	50,802	51.01%	26,683	53.46%	12,978	57.82%		126,945	52.64%
1–2	1498	14.87%	8590	14.53%	14,609	14.67%	7041	14.11%	3165	14.10%		34,903	14.47%
3–4	1142	11.33%	7141	12.08%	12,414	12.46%	5860	11.74%	2250	10.02%		28,807	11.95%
5–6	835	8.29%	5205	8.80%	9001	9.04%	4098	8.21%	1481	6.60%		20,620	8.55%
7	1141	11.32%	7159	12.11%	12,769	12.82%	6233	12.49%	2571	11.45%		29,873	12.39%
Alcohol consumption, times/week											< 0.001		
0	7676	76.17%	40,734	68.91%	62,821	63.08%	31,182	62.47%	14,365	64.00%		156,778	67.40%
< 1	1146	11.37%	7279	12.31%	12,906	12.96%	5790	11.60%	2225	9.91%		29,346	12.62%
1–2	860	8.53%	7531	12.74%	16,077	16.14%	8219	16.47%	3449	15.37%		36,136	15.54%
3–4	221	2.19%	1948	3.30%	4433	4.45%	2517	5.04%	1217	5.42%		10,336	4.44%
≥ 5	174	1.73%	1624	2.75%	3358	3.37%	2207	4.42%	1189	5.30%			0.00%
Household income, quartiles											< 0.001		
First (highest)	3708	36.80%	21,254	35.95%	35,335	35.48%	16,743	33.54%	6866	30.59%		83,906	34.79%
Second	2470	24.51%	14,685	24.84%	24,505	24.60%	12,246	24.53%	5435	24.21%		59,341	24.61%
Third	1920	19.05%	11,488	19.43%	19,105	19.18%	10,208	20.45%	4822	21.48%		47,543	19.72%
Fourth (lowest)	1979	19.64%	11,689	19.77%	20,650	20.73%	10,718	21.47%	5322	23.71%		50,358	20.88%
Fasting blood glucose, mean (SD), mg/dL	114.52 (43.59)		117.74 (43.57)		117.80 (41.65)		119.55 (42.73)		123.60 (47.00)		< 0.001	118.55 (42.99)	
< 100.0	4884	48.47%	24,654	41.70%	39,129	39.29%	18,280	36.62%	7515	33.48%		94,462	39.17%
100.0–125.9	2736	27.15%	18,458	31.22%	33,650	33.79%	17,310	34.68%	7567	33.71%		79,721	33.06%
≥ 126.0	2457	24.38%	16,004	27.07%	26,816	26.93%	14,325	28.70%	7363	32.80%		66,965	27.77%
Total cholesterol, mean (SD), mg/dL	186.85 (39.11)		190.71 (41.34)		193.54 (42.84)		196.51 (46.45)		199.20 (47.34)		< 0.001	193.71 (43.64)	
< 200.0	6615	65.64%	36,604	61.92%	59,020	59.26%	28,087	56.27%	12,023	53.57%		142,349	59.03%
200.0–239.9	2530	25.11%	16,234	27.46%	28,763	28.88%	15,238	30.53%	7005	31.21%		69,770	28.93%
≥ 240.0	932	9.25%	6278	10.62%	11,812	11.86%	6590	13.20%	3417	15.22%		29,029	12.04%
Aspirin											< 0.001		
No	7351	72.95%	42,226	71.43%	70,117	70.40%	35,113	70.35%	15,832	70.54%		170,639	70.76%
Yes	2726	27.05%	16,890	28.57%	29,478	29.60%	14,802	29.65%	6613	29.46%		70,509	29.24%
Statin											< 0.001		
No	7979	79.18%	47,773	80.81%	82,682	83.02%	42,381	84.91%	19,298	85.98%		200,113	82.98%
Yes	2098	20.82%	11,343	19.19%	16,913	16.98%	7534	15.09%	3147	14.02%		41,035	17.02%
Aspirin or statin											< 0.001		
No	6217	61.69%	36,379	61.54%	62,035	62.29%	31,623	63.35%	14,373	64.04%		150,627	62.46%
Yes	3860	38.31%	22,737	38.46%	37,560	37.71%	18,292	36.65%	8072	35.96%		90,521	37.54%
Anti-hypertensive medications											< 0.001		
No	6886	68.33%	36,049	60.98%	55,657	55.88%	26,173	52.44%	10,811	48.17%		135,576	56.22%
Yes	3191	31.67%	23,067	39.02%	43,938	44.12%	23,742	47.56%	11,634	51.83%		105,572	43.78%

SBP, systolic blood pressure; DBP, diastolic blood pressure; IQR, interquartile range; SD, standard deviation

### Cardiovascular events according to BP and age categories

A total of 35,396 events occurred during a median follow-up period of 10.0 years. Cardiovascular events according to BP and age categories are shown in Table 2. In the total population, as BP rose, cardiovascular events more frequently occurred: the incidence of cardiovascular events was lowest in the lowest BP group (< 120/70 mmHg) (1212/10,000 person-years), and highest in the highest BP group (≥ 150/100 mmHg) (2293/10,000 person-years). Compared to patients with BP 130–139/80–89 mmHg, the risk of cardiovascular events was significantly lower in those with BP < 120/70 mmHg and BP 120–129/70–79 mmHg with HR of 0.85 (95% CI 0.80–0.90) and HR of 0.94 (95% CI 0.92–0.97), respectively. Compared to patients with BP 130–139/80–89 mmHg, the risk of cardiovascular events was significantly higher in those with BP 140–149/90–99 mmHg and BP ≥ 150/100 mmHg with HR of 1.12 (95% CI 1.09–1.15) and HR of 1.33 (95% CI 1.29–1.37), respectively.

**Table 2:** Cardiovascular events of study subjects according to BP and age categories

Clinical event	< 120/ < 70 mmHg	120–129/70–79 mmHg	130–139/80–89 mmHg	140–149/90–99 mmHg	≥ 150/ ≥ 100 mmHg
Total					
Events	1123	7625	13,764	8188	4696
Person-years	92,622	567,439	983,762	484,528	204,774
Incidence (events/100,000 person-years)	1212	1344	1399	1690	2293
Adjusted HR (95% CI)	0.85 (0.80–0.90)	0.94 (0.92–0.97)	Ref	1.12 (1.09–1.15)	1.33 (1.29–1.37)
< 50 years					
Events	113	951	1763	874	430
Person-years	30,886	167,650	288,016	119,956	39,039
Incidence (events/100,000 person-years)	366	567	612	729	1101
Adjusted HR (95% CI)	0.74 (0.61–0.90)	0.96 (0.89–1.04)	Ref	1.20 (1.10–1.30)	1.79 (1.61–1.99)
50–59 years					
Events	265	1860	3409	1815	877
Person-years	27,949	182,438	318,830	146,385	55,314
Incidence (events/100,000 person-years)	948	1020	1069	1240	1585
Adjusted HR (95% CI)	0.87 (0.77–0.99)	0.94 (0.89–1.00)	Ref	1.16 (1.10–1.23)	1.43 (1.32–1.54)
60–69 years					
Events	425	2999	5361	3301	1916
Person-years	24,115	158,546	276,831	155,068	72,302
Incidence (events/100,000 person-years)	1762	1892	1937	2129	2650
Adjusted HR (95% CI)	0.86 (0.78–0.95)	0.97 (0.92–1.01)	Ref	1.09 (1.05–1.14)	1.32 (1.25–1.39)
≥ 70 years					
Events	320	1815	3231	2198	1473
Person-years	9670	58,803	100,083	63,116	38,117
Incidence (events/100,000 person-years)	3309	3087	3228	3482	3864
Adjusted HR (95% CI)	0.99 (0.88–1.11)	0.95 (0.89–1.00)	Ref	1.08 (1.03–1.14)	1.17 (1.10–1.25)

HR, hazard ratio; CI, confidence interval. BP, blood pressure

**Fig. 2:**
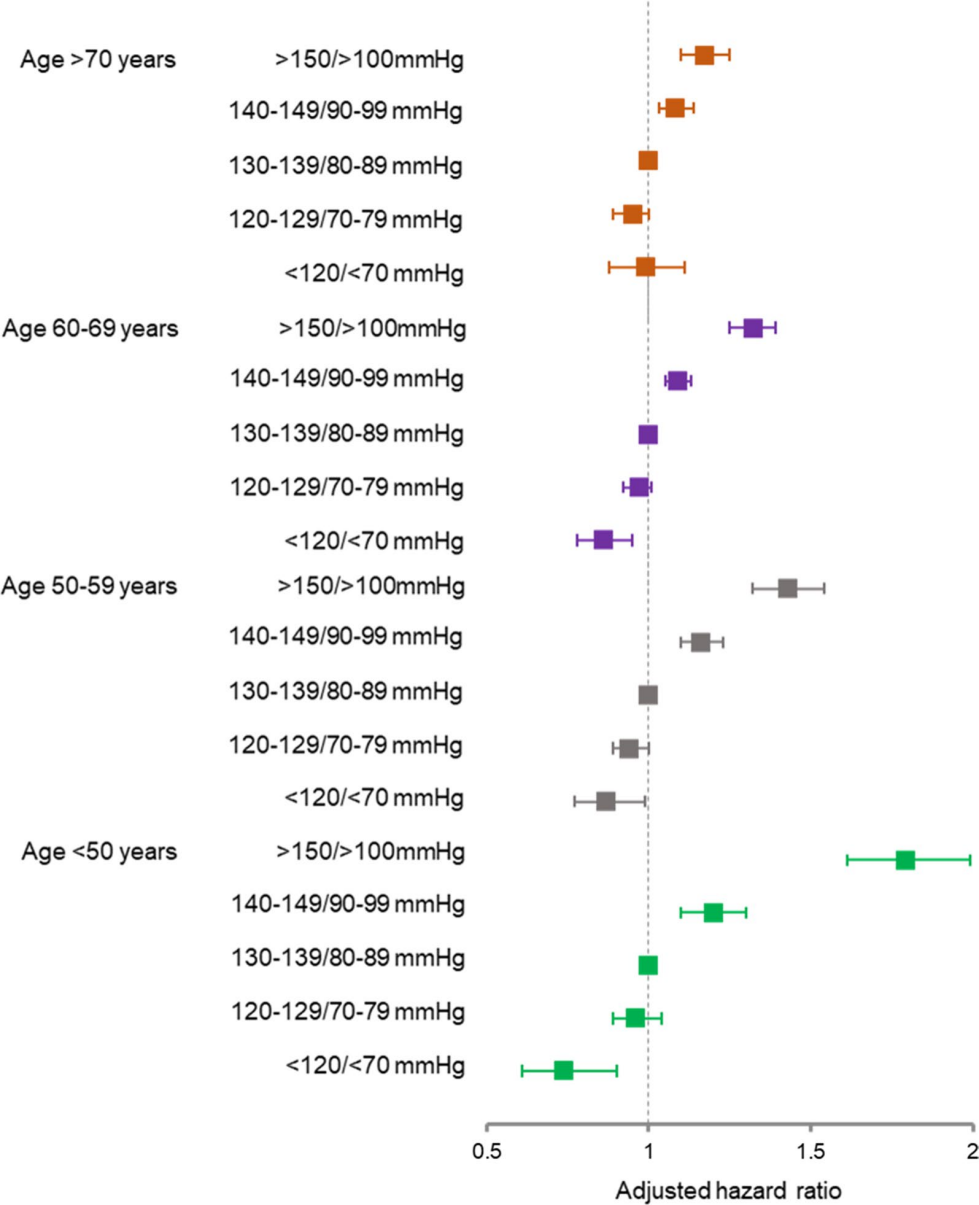
Adjusted hazard ratio for the risk of cardiovascular events according to blood pressure and age categories

**Fig. 3:**
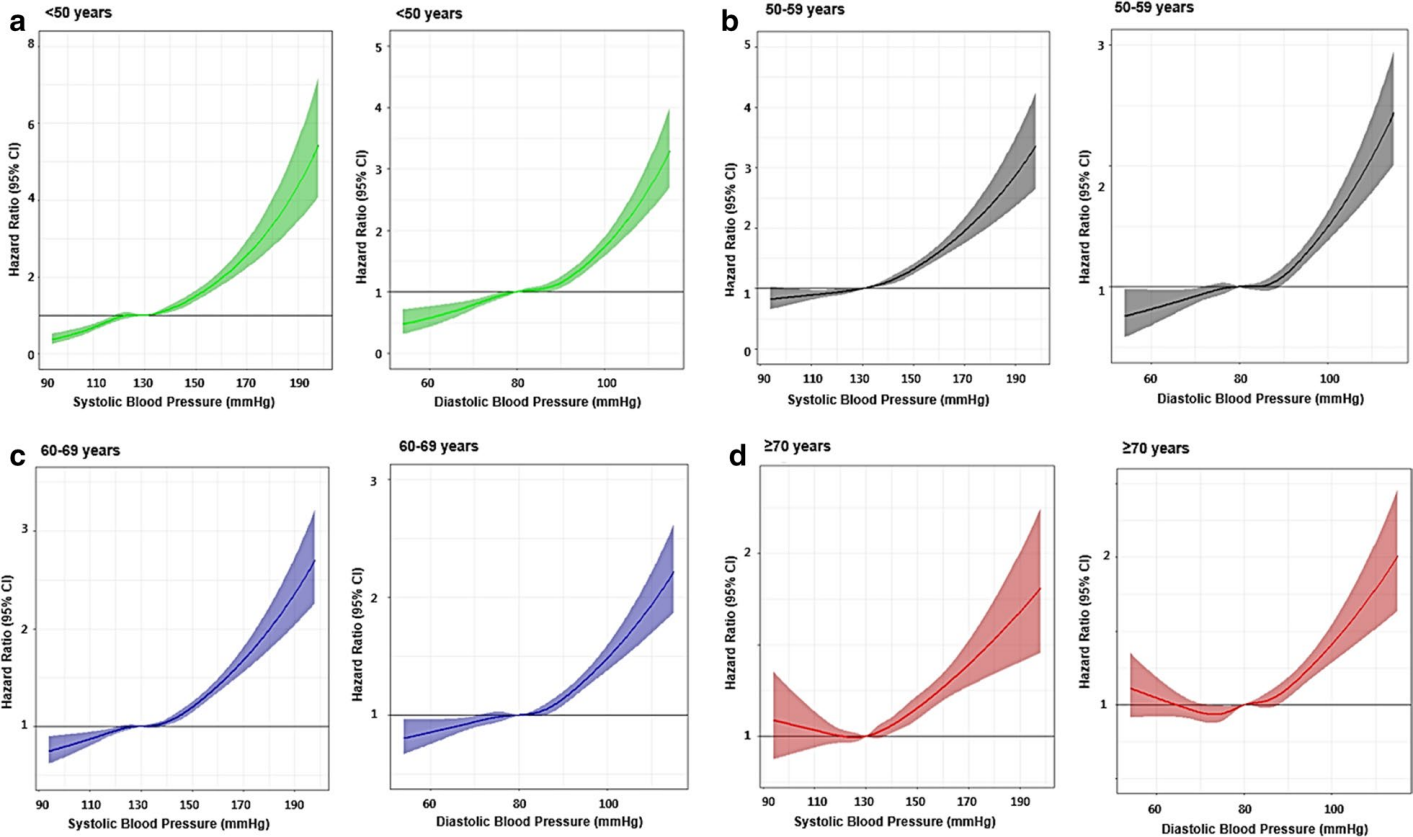
Restricted cubic spline curves showing age-specific adjusted hazard ratios for the risk of cardiovascular events according to SBP and DBP categories. Solid lines indicate hazard ratios and shaded areas indicate 95% confidence intervals. SBP, systolic blood pressure; DBP, diastolic blood pressure

At the age of < 70 years, the risk of cardiovascular events was significantly lower in patients with BP < 120/70 mmHg than in those with BP 130–139/80–89 mmHg. The risk reduction was stronger at younger ages: HRs (95% CIs) were 0.74 (0.61–0.90), 0.87 (0.77–0.99), and 0.86 (0.78–0.95), in patients with < 50 years, 50–59 years, and 60–69 years, respectively. At the age of ≥ 70 years, however, there were no significant differences in the risk of cardiovascular events between patients with BP 130–139/80–89 mmHg and BP < 120/70 mmHg with HR of 0.99 (95% CI 088–1.11). The risk of cardiovascular events was similar between patients with BP 130–139/80–89 mmHg and BP 120–129/70–79 mmHg at all ages. The risk of cardiovascular events was significantly higher in patients with BP ≥ 140/90 mmHg than those with BP 130–139/80–89 mmHg at all ages. The younger the patients, the higher the risk. Adjusted HRs for the risk of cardiovascular events according to BP and age categories are also demonstrated in Fig. 2. Restricted cubic spline curves show age-specific adjusted hazard ratios for cardiovascular events according to systolic BP (SBP) and diastolic BP (DBP) categories (Fig. 3). There was a significant interaction between SBP and age for the prediction of cardiovascular events (interaction *P* < 0.001).

Similar results were obtained in sex-specific analysis (Additional file 1: Tables S1 and S2). Low BP < 120/70 mmHg was more associated with reduced cardiovascular risk in women than in men. The incidence of cardiovascular events with high BP above 130–139/80–89 mmHg was consistently observed regardless of obesity (Additional file 1: Tables S3 and S4). All-cause mortality and primary end-point results according to BP and age categories are demonstrated in Additional file 1: Tables S5–S8. The risk of all-cause or cardiovascular mortality was significantly higher in patients with BP < 120/70 mmHg than in those with BP 130–139/80–89 mmHg in patients with age of ≥ 60 years. The risk of myocardial infarction was not different between patients with BP < 120/70 mmHg and BP 130–139/80–89 mmHg at all ages. However, the risk of stroke was significantly lower in patients with BP < 120/70 mmHg than in those with BP 130–139/80–89 mmHg at all ages. All-cause or cardiovascular mortality and the risk of stroke were significantly higher in patients with BP > 140/90 mmHg than in those with BP 130–139/80–89 mmHg at all ages. The risk of myocardial infarction was significantly increased when BP was ≥ 150/90 mmHg.

### Cardiovascular risks in patients with anti-hypertensive medications

A total of 105,572 (43.8%) patients were taking anti-hypertensive medications. In these patients with anti-hypertensive medications, the risk of cardiovascular events was not different among those with BP < 120/70, 120–129/70–79, and 130–139/80–89 mmHg, and it was significantly higher in patients with BP ≥ 140/90 mmHg than in those with BP 130–139/80–89 mmHg at the age of < 70 years. At the age of ≥ 70 years, the risk of cardiovascular events was not different among patients with BP < 120/70, 120–129/70–79, 130–139/80–89, and 140–149/90–99 mmHg, and it was significantly higher in those with BP ≥ 150/100 mmHg than in those with BP 130–139/80–89 mmHg (Table 3). All-cause and cardiovascular mortality tended to increase in those with BP < 120/70 mmHg, compared to those with BP 130–139/80–89 mmHg at all ages (Additional file 1: Tables S9 and S10). At BP ≥ 150/100 mmHg, the risk of myocardial infarction tended to increase, but the differences were not statistically significant either age (Additional file 1: Table S11). The lower the blood pressure, the lower the risk of stroke at all ages (Additional file 1: Table S12).

**Table 3:** Cardiovascular events of study subjects with anti-hypertensive medications according to BP and age categories

Clinical event	< 120/ < 70 mmHg	120–129/70–79 mmHg	130–139/80–89 mmHg	140–149/90–99 mmHg	≥ 150/ ≥ 100 mmHg
Total					
Events	397	2901	5606	3519	2145
Person-years	25,355	195,990	389,630	20,951	97,809
Incidence (events/100,000 person-years)	1566	1480	1439	16,796	2193
Adjusted HR (95% CI)	0.95 (0.85–1.05)	0.97 (0.93–1.02)	Ref	1.11 (1.06–1.16)	1.29 (1.23–1.36)
< 50 years					
Events	21	214	511	269	136
Person-years	3985	35,306	80,961	38,353	13,671
Incidence (events/100,000 person-years)	527	606	631	701	995
Adjusted HR (95% CI)	0.89 (0.57–1.38)	0.97 (0.82–1.14)	Ref	1.11 (0.95–1.28)	1.58 (1.30–1.91)
50–59 years					
Events	78	586	1285	738	342
Person-years	7829	63,159	127,672	62,997	25,614
Incidence (events/100,000 person-years)	996	928	1006	1171	1335
Adjusted HR (95% CI)	0.97 (0.77–1.22)	0.91 (0.82–1.00)	Ref	1.16 (1.06–1.27)	1.28 (1.13–1.44)
60–69 years					
Events	151	1255	2231	1450	928
Person-years	8827	68,351	128,304	74,550	37,595
Incidence (events/100,000 person-years)	1711	1836	1739	1945	2468
Adjusted HR (95% CI)	0.95 (0.80–1.12)	1.05 (0.98–1.13)	Ref	1.10 (1.03–1.18)	1.37 (1.27–1.48)
≥ 70 years					
Events	147	846	1579	1062	739
Person-years	4713	29,172	52,691	33,611	20,927
Incidence (events/100,000 person-years)	3119	2900	2997	3160	3531
Adjusted HR (95% CI)	1.00 (0.85–1.19)	0.95 (0.87–1.03)	Ref	1.05 (0.97–1.14)	1.15 (1.05–1.25)

HR, hazard ratio; CI, confidence interval. BP, blood pressure

## Discussion

In this nationwide population-based cohort of 241,148 patients with both hypertension and DM, but without CVD, those with BP < 120/70 mmHg had significantly lower risk of cardiovascular events than those with BP 130–139/80–89 mmHg at the age of < 70 years. However, there was no significant difference in the risk of cardiovascular events in patients with BP < 120/70 mmHg and BP 130–139/80–89 mmHg at the age ≥ 70. For patients on anti-hypertensive medications, the risk of cardiovascular events was similar between patients with BP ≤ 130–139/80–89 mmHg and higher in those with BP ≥ 140/90 mmHg at all ages. These results suggest that optimal target BP in patients with DM may differ according to age and that: lowering BP to < 130/80 mmHg may be effective at the age of < 70 years, but not in those at the age of ≥ 70 years. In addition, the lower the better may not be applied in patients on anti-hypertensive medications, because all-cause or cardiovascular mortality tended to be even higher in those with < 120/70 mmHg. Based on these results, the drug goal should be less than 140/90 mmHg but individualized.

Although high prevalence of hypertension in patients with DM [7], and markedly increased risk of cardiovascular events in coexistence of hypertension and DM [8, 9], there is limited data on optimal target BP in patients with DM. In a large randomized controlled trial (RCT) of patients with DM demonstrated that lowering SBP to < 135 mmHg using perindopril and indapamide regimen was shown to be significantly associated with reductions in cardiovascular events, compared to the placebo group whose SBP was maintained at ~ 140 mmHg [19]. However, another RCT showed that, compared with ~ 135 mmHg, an achieved SBP to 121 mmHg did not reduce cardiovascular morbidity and mortality in patients with DM [20]. Meta-analyses confirmed that reduction of SBP of < 140 mmHg is associated with better cardiovascular outcomes [21], but there is no beneficial effect when SBP is lowered to < 130 mmHg in patients with DM [22]. Another meta-analysis of 73,914 subjects with DM reported that lowering SBP to < 130 mmHg recued stroke by 39%; however, there was no risk reduction in myocardial infarction [23]. Excluding the effect of strong glycemic control in diabetic patients, a more intensive lowering SBP to < 130 mmHg improved overall outcomes [24]. On the line of similar results, recent meta-analyses showed that in diabetic patients, if the baseline SBP ≥ 140 mmHg, antihypertensive treatment reduced cardiovascular risk; however, if the baseline SBP < 140 mmHg, there was no observed benefit in BP lowering therapy [25, 26]. As mentioned above, each study has different target BP, and the results are slightly different, making it difficult to clarify where to put the target BP in patients with DM. In a whole study population in our study, the lower the BP, the lower the cardiovascular events, and thus, the target BP of DM may be suggested as < 130/80 mmHg, if we do not consider age. Most of the existing studies, including meta-analysis, were conducted in the West, but this study is an Asian study, and racial differences should be considered when interpreting our results.

However, age should be considered when setting target BP in hypertensive subjects. Although lowering BP obviously improves clinical outcome [12, 13], adverse effects more frequently occur with intensive treatment in older people [14, 27]. There is still no standard guideline for target BP in elderly subjects. The 2 most widely used guidelines show somewhat differences in target BP in older people. The 2017 ACC/AHA guideline recommends a target BP of < 130/80 mmHg in the elderly, which is the same in younger age [15]. However, there is disagreement with setting the same target BP (< 130/80 mmHg) in subjects aged 30 and 80 years [27]. Indeed, lowering BP to < 130/80 mmHg is difficult in some elderly subjects, especially when they have isolated systolic hypertension and poor vascular compliance [27]. Also, there is concern about more frequent and serious adverse effects from intensive BP control in more frail older subjects [13]. In this context, the 2018 European Society of Cardiology/European Society of Hypertension (ESC/ESH) guideline recommended that in older subjects on BP-lowering drugs, BP should be lowered to < 140/80 mmHg, but not SBP < 130 mmHg [16]. Although age is an important factor for hypertension control, there have been few studies on whether age should be considered when setting target BP in subjects with DM. In the present study, the risk of cardiovascular events was not different among patients with BP < 120/70, 120–129/70–79 and 130–139/80–89 mmHg at the age of ≥ 70 years, suggesting that older subjects with DM do not need strict BP control, which is in line with 2018 ESC/ESH guideline [16]. For patients on anti-hypertensive medications, our study showed that the risk of cardiovascular events was similar in all patients with BP ≤ 130–139/80–89 mmHg and higher in those with BP ≥ 140/90 mmHg, at all ages. Rather, when BP was lowered to < 130/80 mmHg, all-cause or cardiovascular mortality tended to rise at all ages. These results suggesting different target BPs according to age and anti-hypertensive medications in diabetic patients deserve attention and could be of clinical use.

The results of the present study showed that the risk of cardiovascular events associated with elevated BP decreased as patients became older: reduction in BP from 130–139/80–89 to < 120/70 mmHg was associated 26% reduction in the risk of cardiovascular events at the age of < 50 years, as low as 13% ~ 14% at the age of 50–69 years, and no beneficial effect at the age of ≥ 70 years. These results are in line with those of previous studies in the general population [28]. In the elderly, irreversible pathological changes in vasculature caused by long-standing high BP may develop and lead to cardiovascular events despite a lowered, even normalized BP [27]. The effective prevention of cardiovascular events can be expected by lowering BP in younger patients with DM, so that they need more strict BP control.

Our results suggest that BP can be safely lowered to < 130/80 mmHg in younger patients with DM, but not in elderly diabetics aged ≥ 70 years. However, current study analyzed diabetic patients who were relatively healthy, had no history of CVD and received regular health check-ups. In high-risk diabetics with a history of CVD, the target BP of < 130/80 mmHg may be more appropriate even at the age of ≥ 70 years [29, 30]. Otherwise, target BP should not be lowered to < 130/80 mmHg in diabetic patients with comorbidities and high frailty even at the age of < 70 years due to the risk of side effects of intensive BP lowering [31]. Target BP should be individualized according to the risk of cardiovascular events, comorbidities, frailty and age [32]. In addition, the lower the better is not applied in patients on anti-hypertensive medications: at BP < 140/90 mmHg, there were no differences in the risk of cardiovascular events, but all-cause and cardiovascular mortality was significantly increased at BP < 120/70 mmHg at all ages. Based on these results, the target goal of anti-hypertensive medications should be BP < 140/90 mmHg, but not BP < 120/70 mmHg.

## Limitations

Besides inherent shortcomings of the use of administrative database and retrospective design, there are several limitations to this study. First, as the diagnosis of hypertension in our study was based on the diagnostic code, not on the BP levels, there are several possible reasons why many patients diagnosed with hypertension had relatively normal or even low BP, even though many patients were not taking anti-hypertensive medications, as follows: (1) there might be coding errors which have always been an issue when using claim data. Nevertheless, it is reported that the diagnosis accuracy of hypertension is relatively high in claim data (sensitivity = 73% and positive predictive value = 82%) [33], (2) patients who were prescribed anti-hypertensive medications along with health check-ups at regular basis were classified as those who take anti-hypertensive medications in the current study. Thus, it was possible that some patients who were prescribed anti-hypertensive medications on an irregular basis or who did not undergo health check-ups would be miss-classified as that they were not taking anti-hypertensive medications even though they were consistently taking the medications, and (3) we used average value of two would be lower than expected. Despite the various shortcomings, claim data has a strength in that it is not limited to a specific doctors or specific medical institutions, and there is no selection bias. In addition, only patients taking anti-hypertensive medications (they might be certain to be hypertensive) were analyzed separately, and we showed the same results as all patients. Second, as clinic BP measurements were made for the analysis, BP values might be less accurate. BP values from out-of-office BP monitoring such as ambulatory blood pressure monitoring or home blood pressure monitoring may provide more valuable information [34]. In order to minimize errors and inaccuracies, we averaged 2 measurements of BP. Third, the side effects of lowering BP were not identified in this study. Fourth, as the results of our study were obtained from relatively healthy diabetic patients without CVD, it should be noted that it is difficult to apply our results directly to high-risk patients with CVD or those with comorbidity and high frailty [29, 31]. Fifth, we need to be careful when interpreting the results for the secondary study endpoint of the study. In the analysis of each clinical event (the secondary study endpoint, represented in Additional file 1), the incidence of clinical event in each group was very low, so the statistical power would have been weakened. Sixth, information on anti-diabetic medications was not available in our study, because some important anti-diabetic drugs such as dipeptidyl peptidase-4 inhibitors and sodium-glucose co-transporter-2 inhibitors were introduced into the domestic market after patients’ enrollment. Lastly, our results were obtained from all Korean patients, so that its application to other ethnic groups may be limited.

## Conclusion

In a cohort of hypertensive patients who had DM but no history of CVD, lower BP was associated with lower risk of cardiovascular events especially at the age of < 70 years. Effort to lower BP of < 130/80 mmHg may be justified in patients at the age of < 70 years; however, the intensive BP lowering strategy is less beneficial in those at the age of ≥ 70 years, and lowering BP to 130–139/80–89 mmHg would be appropriate at the age of ≥ 70. In patients on anti-hypertensive medications, target BP should be < 140/90 mmHg, but not BP < 120/70 mmHg. Well-designed prospective studies are needed to verify our findings.

## Supplementary information

**Additional file 1: Table S1.** Incidence of primary end-point* according to BP and age categories in men. **Table S2.** Incidence of primary end-point* according to BP and age categories in women. **Table S3.** Incidence of primary end-point* according to BP and age categories in patients with body mass index ≥ 25 kg/m^2^. **Table S4.** Incidence of primary end-point* according to BP and age categories in patients with body mass index < 25 kg/m^2^. **Table S5.** All-cause mortality according BP and age categories. **Table S6.** Cardiovascular mortality according to BP and age categories. **Table S7.** Incidence of myocardial infarction according to BP and age categories. **Table S8.** Incidence of stroke according to BP and age categories. **Table S9.** All-cause mortality of study subjects with anti-hypertensive medications according to BP and age categories. **Table S10.** Cardiovascular mortality of study subjects with anti-hypertensive medications according to BP and age categories. **Table S11.** Incidence of myocardial infarction of study subjects with anti-hypertensive medications according to BP and age categories. **Table S12.** Incidence of stroke of study subjects with anti-hypertensive medications according to BP and age categories.

## Data Availability

The datasets used and analyzed during the current study are available from the corresponding author on reasonable request.

## Abbreviations

BP: Blood pressure
SBP: Systolic blood pressure
DBP: Diastolic blood pressure
DM: Diabetes mellitus
CVD: Cardiovascular disease
ACC/AHA: American College of Cardiology/American Heart Association
ESC/ESH: European Society of Cardiology/European Society of Hypertension
NIHS-HEALS: National Health Insurance Services-Health Screening
RCT: Randomized controlled trial
