# Supplementary material for: Blood pressure levels and cardiovascular risk according to age in patients with diabetes mellitus: a nationwide population-based cohort study
Source: Cardiovasc Diabetol. 2020 Oct 19;19:181. doi: 10.1186/s12933-020-01156-8 (PMC7574489; doi:10.1186/s12933-020-01156-8)
Supplement: Supplementary file 1 — Additional file 1: Table S1. Incidence of primary end-point* according to BP and age categories in men. Table S2. Incidence of primary end-point* according to BP and age categories in women. Table S3. Incidence of primary end-point* according to BP and age categories in patients with body mass index ≥ 25 kg/m2. Table S4. Incidence of primary end-point* according to BP and age categories in patients with body mass index < 25 kg/m2. Table S5. All-cause mortality according BP and age categories. Table S6. Cardiovascular mortality according to BP and age categories. Table S7. Incidence of myocardial infarction according to BP and age categories. Table S8. Incidence of stroke according to BP and age categories. Table S9. All-cause mortality of study subjects with anti-hypertensive medications according to BP and age categories. Table S10. Cardiovascular mortality of study subjects with anti-hypertensive medications according to BP and age categories. Table S11. Incidence of myocardial infarction of study subjects with anti-hypertensive medications according to BP and age categories. Table S12. Incidence of stroke of study subjects with anti-hypertensive medications according to BP and age categories. [file 12933_2020_1156_MOESM1_ESM.docx]

**Supplementary Table S1. Incidence of primary end-point^*^ according to BP and age categories in men**

| **Clinical event** | **<120/<70 mmHg** | **120-129/70-79 mmHg** | **130-139/80-89 mmHg** | **140-149/90-99 mmHg** | **≥150/≥100 mmHg** |
| --- | --- | --- | --- | --- | --- |
| **Total** |  |  |  |  |  |
| Events | 551 | 3947 | 7564 | 4371 | 2421 |
| Person-years | 33138 | 274717 | 546768 | 266676 | 107689 |
| Incidence (events/100000 person-years) | 1663 | 1437 | 1383 | 1639 | 2248 |
| Adjusted HR (95% CI) | 0.94 (0.87-1.03) | 0.97 (0.94-1.01) | Ref. | 1.12 (1.08-1.16) | 1.33 (1.27-1.40) |
| **<50 years** |  |  |  |  |  |
| Events | 62 | 694 | 1415 | 707 | 348 |
| Person-years | 10093 | 102876 | 212162 | 91275 | 29847 |
| Incidence (events/100000 person-years) | 614 | 675 | 667 | 775 | 1166 |
| Adjusted HR (95% CI) | 0.82 (0.64-1.06) | 0.97 (0.89-1.06) | Ref. | 1.18 (1.80-1.29) | 1.76 (1.56-1.98) |
| **50-59 years** |  |  |  |  |  |
| Events | 139 | 1096 | 2229 | 1160 | 570 |
| Person-years | 9785 | 85148 | 175697 | 82873 | 30932 |
| Incidence (events/100000 person-years) | 1421 | 1287 | 1269 | 1400 | 1843 |
| Adjusted HR (95% CI) | 0.95 (0.80-1.13) | 0.95 (0.89-1.02) | Ref. | 1.12 (1.04-1.20) | 1.41 (1.28-1.54) |
| **60-69 years** |  |  |  |  |  |
| Events | 206 | 1438 | 2657 | 1596 | 958 |
| Person-years | 9628 | 65332 | 122491 | 69907 | 33129 |
| Incidence (events/100000 person-years) | 2140 | 2201 | 2169 | 2283 | 2892 |
| Adjusted HR (95% CI) | 0.89 (0.77-1.02) | 0.99 (0.92-1.05) | Ref. | 1.06 (1.00-1.13) | 1.31 (1.21-1.41) |
| **≥70 years** |  |  |  |  |  |
| Events | 144 | 719 | 1263 | 908 | 545 |
| Person-years | 3630 | 21360 | 36417 | 22618 | 13780 |
| Incidence (events/100000 person-years) | 3967 | 3366 | 3468 | 4015 | 3955 |
| Adjusted HR (95% CI) | 1.09 (0.91-1.29) | 0.95 (0.87-1.05) | Ref. | 1.16 (1.07-1.27) | 1.12 (1.02-1.24) |

^*^Cardiovascular death, myocardial infarction and stroke. HR, hazard ratio; CI, confidence interval. BP, blood pressure.

**Supplementary Table S2. Incidence of primary end-point^*^ according to BP and age categories in women**

| **Clinical event** | **<120/<70 mmHg** | **120-129/70-79 mmHg** | **130-139/80-89 mmHg** | **140-149/90-99 mmHg** | **≥150/≥100 mmHg** |
| --- | --- | --- | --- | --- | --- |
| **Total** |  |  |  |  |  |
| Events | 572 | 3678 | 6200 | 3817 | 2275 |
| Person-years | 59483 | 292722 | 436994 | 217852 | 97085 |
| Incidence (events/100000 person-years) | 962 | 1256 | 1419 | 1752 | 2343 |
| Adjusted HR (95% CI) | 0.82 (0.76-0.90) | 0.94 (0.90-0.98) | Ref. | 1.12 (1.08-1.17) | 1.31 (1.25-1.38) |
| **<50 years** |  |  |  |  |  |
| Events | 51 | 257 | 348 | 167 | 82 |
| Person-years | 20792 | 64774 | 75854 | 28681 | 9191 |
| Incidence (events/100000 person-years) | 245 | 397 | 459 | 582 | 892 |
| Adjusted HR (95% CI) | 0.65 (0.48-0.88) | 0.92 (0.78-1.08) | Ref. | 1.25 (1.04-1.50) | 1.86 (1.46-2.37) |
| **50-59 years** |  |  |  |  |  |
| Events | 126 | 764 | 1180 | 655 | 307 |
| Person-years | 18163 | 97290 | 143133 | 63511 | 24382 |
| Incidence (events/100000 person-years) | 694 | 785 | 824 | 1031 | 1259 |
| Adjusted HR (95% CI) | 0.81 (0.67-0.97) | 0.93 (0.85-1.02) | Ref. | 1.23 (1.12-1.36) | 1.46 (1.29-1.66) |
| **60-69 years** |  |  |  |  |  |
| Events | 219 | 1561 | 2704 | 1705 | 958 |
| Person-years | 14486 | 93214 | 154340 | 85161 | 39173 |
| Incidence (events/100000 person-years) | 1512 | 1675 | 1752 | 2002 | 2446 |
| Adjusted HR (95% CI) | 0.84 (0.73-0.96) | 0.95 (0.89-1.01) | Ref. | 1.13 (1.06-1.20) | 1.34 (1.24-1.44) |
| **≥70 years** |  |  |  |  |  |
| Events | 176 | 1096 | 1968 | 1290 | 928 |
| Person-years | 6040 | 37442 | 63665 | 40497 | 24336 |
| Incidence (events/100000 person-years) | 2914 | 2927 | 3091 | 3185 | 3813 |
| Adjusted HR (95% CI) | 0.92 (0.79-1.07) | 0.94 (0.88-1.02) | Ref. | 1.04 (0.96-1.10) | 1.20 (1.11-1.30) |

^*^Cardiovascular death, myocardial infarction and stroke. BP, blood pressure; HR, hazard ratio; CI, confidence interval.

**Supplementary Table S3. Incidence of primary end-point^*^ according to BP and age categories in patients with body mass index ≥25 kg/m^2^**

| **Clinical event** | **<120/<70 mmHg** | **120-129/70-79 mmHg** | **130-139/80-89 mmHg** | **140-149/90-99 mmHg** | **≥150/≥100 mmHg** |
| --- | --- | --- | --- | --- | --- |
| **Total** |  |  |  |  |  |
| Events | 291 | 2824 | 6190 | 3862 | 2164 |
| Person-years | 24795 | 233515 | 492155 | 255980 | 106568 |
| Incidence (events/100000 person-years) | 1174 | 1209 | 1258 | 1509 | 2031 |
| Adjusted HR (95% CI) | 0.87 (0.77-0.97) | 0.94 (0.90-0.98) | Ref. | 1.11 (1.07-1.16) | 1.34 (1.28-1.41) |
| **<50 years** |  |  |  |  |  |
| Events | 37 | 400 | 911 | 500 | 248 |
| Person-years | 6386 | 68933 | 157271 | 71721 | 24100 |
| Incidence (events/100000 person-years) | 579 | 580 | 579 | 697 | 1029 |
| Adjusted HR (95% CI) | 1.10 (0.79-1.53) | 1.02 (0.91-1.15) | Ref. | 1.22 (1.09-1.36) | 1.81 (1.57-2.09) |
| **50-59 years** |  |  |  |  |  |
| Events | 65 | 735 | 1660 | 903 | 454 |
| Person-years | 8942 | 79307 | 163233 | 79741 | 30631 |
| Incidence (events/100000 person-years) | 727 | 927 | 1017 | 1132 | 1482 |
| Adjusted HR (95% CI) | 0.72 (0.56-0.93) | 0.91 (0.84-1.00) | Ref. | 1.11 (1.03-1.21) | 1.44 (1.29-1.59) |
| **60-69 years** |  |  |  |  |  |
| Events | 109 | 1139 | 2427 | 1585 | 914 |
| Person-years | 6879 | 65179 | 133045 | 78492 | 36789 |
| Incidence (events/100000 person-years) | 1585 | 1747 | 1824 | 2019 | 2484 |
| Adjusted HR (95% CI) | 0.85 (0.70-1.03) | 0.96 (0.89-1.03) | Ref. | 1.09 (1.02-1.16) | 1.32 (1.22-1.42) |
| **≥70 years** |  |  |  |  |  |
| Events | 80 | 550 | 1192 | 874 | 548 |
| Person-years | 2586 | 20095 | 38605 | 26024 | 15047 |
| Incidence (events/100000 person-years) | 3094 | 2737 | 3088 | 3358 | 3642 |
| Adjusted HR (95% CI) | 1.00 (0.80-1.25) | 0.88 (0.80-0.98) | Ref. | 1.07 (0.98-1.17) | 1.13 (1.02-1.25) |

^*^Cardiovascular death, myocardial infarction and stroke. HR, hazard ratio; CI, confidence interval. BP, blood pressure.

**Supplementary Table S4. Incidence of primary end-point^*^ according to BP and age categories in patients with body mass index <25 kg/m^2^**

| **Clinical event** | **<120/<70 mmHg** | **120-129/70-79 mmHg** | **130-139/80-89 mmHg** | **140-149/90-99 mmHg** | **≥150/≥100 mmHg** |
| --- | --- | --- | --- | --- | --- |
| **Total** |  |  |  |  |  |
| Events | 832 | 4801 | 7574 | 4326 | 2532 |
| Person-years | 67825 | 333923 | 491606 | 228546 | 98205 |
| Incidence (events/100000 person-years) | 1227 | 1438 | 1541 | 1893 | 2578 |
| Adjusted HR (95% CI) | 0.87 (0.81-0.93) | 0.96 (0.93-1.00) | Ref. | 1.13 (1.08-1.17) | 1.31 (1.25-1.37) |
| **<50 years** |  |  |  |  |  |
| Events | 76 | 551 | 825 | 374 | 182 |
| Person-years | 24499 | 98716 | 130744 | 48234 | 14938 |
| Incidence (events/100000 person-years) | 310 | 558 | 631 | 775 | 1218 |
| Adjusted HR (95% CI) | 0.64 (0.51-0.82) | 0.91 (0.81-1.01) | Ref. | 1.17 (1.04-1.33) | 1.75 (1.49-2.06) |
| **50-59 years** |  |  |  |  |  |
| Events | 200 | 1125 | 1749 | 912 | 423 |
| Person-years | 19006 | 103131 | 155597 | 66644 | 24683 |
| Incidence (events/100000 person-years) | 1052 | 1091 | 1124 | 1368 | 1714 |
| Adjusted HR (95% CI) | 0.95 (0.82-1.10) | 0.97 (0.90-1.04) | Ref. | 1.20 (1.11-1.30) | 1.42 (1.27-1.58) |
| **60-69 years** |  |  |  |  |  |
| Events | 316 | 1860 | 2934 | 1716 | 1002 |
| Person-years | 17235 | 93367 | 143786 | 76576 | 35513 |
| Incidence (events/100000 person-years) | 1833 | 1992 | 2041 | 2241 | 2822 |
| Adjusted HR (95% CI) | 0.87 (0.77-0.98) | 0.97 (0.92-1.03) | Ref. | 1.10 (1.03-1.17) | 1.32 (1.23-1.42) |
| **≥70 years** |  |  |  |  |  |
| Events | 240 | 1265 | 2039 | 1324 | 925 |
| Person-years | 7083 | 38707 | 61477 | 37091 | 23070 |
| Incidence (events/100000 person-years) | 3388 | 3268 | 3317 | 3570 | 4010 |
| Adjusted HR (95% CI) | 0.99 (0.86-1.13) | 0.98 (0.91-1.05) | Ref. | 1.08 (1.01-1.16) | 1.19 (1.10-1.29) |

^*^Cardiovascular death, myocardial infarction and stroke. BP, blood pressure; HR, hazard ratio; CI, confidence interval.

**Supplementary Table S5. All-cause mortality according BP and age categories**

| **Clinical event** | **<120/<70 mmHg** | **120-129/70-79 mmHg** | **130-139/80-89 mmHg** | **140-149/90-99 mmHg** | **≥150/≥100 mmHg** |
| --- | --- | --- | --- | --- | --- |
| **Total** |  |  |  |  |  |
| Events | 1194 | 6395 | 1119 | 7112 | 4620 |
| Person-years | 93848 | 587630 | 1023804 | 504441 | 211976 |
| Incidence (events/100000 person-years) | 1272 | 1088 | 109 | 1410 | 2179 |
| Adjusted HR (95% CI) | 1.15 (1.09-1.23) | 0.99 (0.96-1.02) | Ref. | 1.14 (1.10-1.17) | 1.42 (1.37-1.47) |
| **<50 years** |  |  |  |  |  |
| Events | 72 | 452 | 761 | 442 | 258 |
| Person-years | 31176 | 171720 | 296074 | 123315 | 40117 |
| Incidence (events/100000 person-years) | 231 | 263 | 257 | 358 | 643 |
| Adjusted HR (95% CI) | 1.16 (0.90-1.48) | 1.07 (0.95-1.21) | Ref. | 1.35 (1.20-1.51) | 2.23 (1.93-2.57) |
| **50-59 years** |  |  |  |  |  |
| Events | 173 | 980 | 1889 | 1075 | 641 |
| Person-years | 28755 | 190308 | 333312 | 153215 | 57921 |
| Incidence (events/100000 person-years) | 602 | 515 | 567 | 702 | 1107 |
| Adjusted HR (95% CI) | 1.13 (0.97-1.32) | 0.92 (0.85-1.00) | Ref. | 1.20 (1.12-1.30) | 1.77 (1.62-1.94) |
| **60-69 years** |  |  |  |  |  |
| Events | 476 | 2418 | 4252 | 2652 | 1697 |
| Person-years | 24593 | 167147 | 293182 | 164188 | 76324 |
| Incidence (events/100000 person-years) | 1936 | 1447 | 1450 | 1615 | 2223 |
| Adjusted HR (95% CI) | 1.24 (1.13-1.36) | 0.99 (0.94-1.04) | Ref. | 1.09 (1.04-1.15) | 1.44 (1.36-1.53) |
| **≥70 years** |  |  |  |  |  |
| Events | 473 | 2545 | 4217 | 2943 | 2024 |
| Person-years | 9322 | 58453 | 101235 | 63821 | 37612 |
| Incidence (events/100000 person-years) | 5074 | 4354 | 4166 | 4611 | 5381 |
| Adjusted HR (95% CI) | 1.17 (1.06-1.28) | 1.05 (1.00-1.10) | Ref. | 1.10 (1.05-1.15) | 1.22 (1.16-1.29) |

BP, blood pressure; HR, hazard ratio; CI, confidence interval.

**Supplementary Table S6. Cardiovascular mortality according to BP and age categories**

| **Clinical event** | **<120/<70 mmHg** | **120-129/70-79 mmHg** | **130-139/80-89 mmHg** | **140-149/90-99 mmHg** | **≥150/≥100 mmHg** |
| --- | --- | --- | --- | --- | --- |
| **Total** |  |  |  |  |  |
| Events | 287 | 1452 | 2527 | 1761 | 1257 |
| Person-years | 93848 | 587630 | 1023804 | 504441 | 211976 |
| Incidence (events/100000 person-years) | 306 | 247 | 247 | 349 | 593 |
| Adjusted HR (95% CI) | 1.23 (1.09-1.39) | 0.99 (0.93-1.06) | Ref. | 1.22 (1.15-1.30) | 1.66 (1.55-1.77) |
| **<50 years** |  |  |  |  |  |
| Events | 16 | 57 | 156 | 96 | 71 |
| Person-years | 31176 | 171720 | 296074 | 123315 | 40117 |
| Incidence (events/100000 person-years) | 51 | 33 | 53 | 78 | 177 |
| Adjusted HR (95% CI) | 1.28 (0.75-2.17) | 0.65 (0.48-0.89) | Ref. | 1.47 (1.14-1.90) | 3.18 (2.39-4.23) |
| **50-59 years** |  |  |  |  |  |
| Events | 28 | 207 | 339 | 199 | 142 |
| Person-years | 28755 | 190308 | 333312 | 153215 | 57921 |
| Incidence (events/100000 person-years) | 97 | 109 | 102 | 130 | 245 |
| Adjusted HR (95% CI) | 1.06 (0.72-1.56) | 1.10 (0.93-1.31) | Ref. | 1.25 (1.05-1.49) | 2.20 (1.81-2.68) |
| **60-69 years** |  |  |  |  |  |
| Events | 106 | 574 | 939 | 683 | 450 |
| Person-years | 24593 | 167147 | 293182 | 164188 | 76324 |
| Incidence (events/100000 person-years) | 431 | 343 | 320 | 416 | 590 |
| Adjusted HR (95% CI) | 1.25 (1.02-1.53) | 1.06 (0.96-1.18) | Ref. | 1.28 (1.16-1.41) | 1.75 (1.56-1.96) |
| **≥70 years** |  |  |  |  |  |
| Events | 137 | 614 | 1093 | 783 | 594 |
| Person-years | 9322 | 58453 | 101235 | 63821 | 37612 |
| Incidence (events/100000 person-years) | 1470 | 1050 | 1080 | 1227 | 1579 |
| Adjusted HR (95% CI) | 1.32 (1.11-1.58) | 0.98 (0.89-1.08) | Ref. | 1.12 (1.02-1.23) | 1.37 (1.24-1.51) |

BP, blood pressure; HR, hazard ratio; CI, confidence interval.

**Supplementary Table S7. Incidence of myocardial infarction according to BP and age categories**

| **Clinical event** | **<120/<70 mmHg** | **120-129/70-79 mmHg** | **130-139/80-89 mmHg** | **140-149/90-99 mmHg** | **≥150/≥100 mmHg** |
| --- | --- | --- | --- | --- | --- |
| **Total** |  |  |  |  |  |
| Events | 211 | 1558 | 2600 | 1356 | 734 |
| Person-years | 98028 | 605393 | 1055590 | 529388 | 231133 |
| Incidence (events/100000 person-years) | 215 | 257 | 246 | 256 | 318 |
| Adjusted HR (95% CI) | 0.86 (0.75-0.99) | 1.02 (0.96-1.09) | Ref. | 1.02 (0.95-1.09) | 1.18 (1.09-1.29) |
| **<50 years** |  |  |  |  |  |
| Events | 23 | 300 | 515 | 228 | 69 |
| Person-years | 31432 | 171895 | 296218 | 124249 | 41316 |
| Incidence (events/100000 person-years) | 73 | 175 | 174 | 184 | 167 |
| Adjusted HR (95% CI) | 0.66 (0.43-1.00) | 1.09 (0.94-1.26) | Ref. | 1.07 (0.92-1.25) | 1.00 (0.78-1.29) |
| **50-59 years** |  |  |  |  |  |
| Events | 63 | 431 | 755 | 344 | 160 |
| Person-years | 29165 | 191707 | 336833 | 156559 | 60287 |
| Incidence (events/100000 person-years) | 216 | 225 | 224 | 220 | 265 |
| Adjusted HR (95% CI) | 1.00 (0.77-1.30) | 1.01 (0.90-1.14) | Ref. | 1.00 (0.88-1.14) | 1.20 (1.01-1.42) |
| **60-69 years** |  |  |  |  |  |
| Events | 72 | 569 | 892 | 517 | 294 |
| Person-years | 26196 | 173663 | 305677 | 172859 | 83087 |
| Incidence (events/100000 person-years) | 275 | 328 | 292 | 299 | 354 |
| Adjusted HR (95% CI) | 0.88 (0.69-1.12) | 1.11 (0.99-1.23) | Ref. | 1.04 (0.93-1.16) | 1.21 (1.06-1.38) |
| **≥70 years** |  |  |  |  |  |
| Events | 53 | 258 | 438 | 267 | 211 |
| Person-years | 11233 | 68126 | 116861 | 75719 | 46442 |
| Incidence (events/100000 person-years) | 472 | 379 | 375 | 353 | 454 |
| Adjusted HR (95% CI) | 1.19 (0.87-1.58) | 0.99 (0.85-1.16) | Ref. | 0.94 (0.81-1.09) | 1.19 (1.01-1.41) |

BP, blood pressure; HR, hazard ratio; CI, confidence interval.

**Supplementary Table S8. Incidence of stroke according to BP and age categories**

| **Clinical event** | **<120/<70 mmHg** | **120-129/70-79 mmHg** | **130-139/80-89 mmHg** | **140-149/90-99 mmHg** | **≥150/≥100 mmHg** |
| --- | --- | --- | --- | --- | --- |
| **Total** |  |  |  |  |  |
| Events | 750 | 5441 | 10103 | 6134 | 3449 |
| Person-years | 94720 | 579947 | 1005224 | 496102 | 212153 |
| Incidence (events/100000 person-years) | 792 | 938 | 1005 | 1236 | 1626 |
| Adjusted HR (95% CI) | 0.77 (0.72-0.83) | 0.92 (0.89-0.95) | Ref. | 1.14 (1.11-1.18) | 1.31 (1.26-1.36) |
| **<50 years** |  |  |  |  |  |
| Events | 78 | 625 | 1176 | 604 | 321 |
| Person-years | 31103 | 169784 | 291884 | 121623 | 39714 |
| Incidence (events/100000 person-years) | 251 | 368 | 403 | 497 | 808 |
| Adjusted HR (95% CI) | 0.70 (0.55-0.88) | 0.93 (0.84-1.02) | Ref. | 1.23 (1.12-1.36) | 1.96 (1.73-2.22) |
| **50-59 years** |  |  |  |  |  |
| Events | 185 | 1333 | 2492 | 1392 | 665 |
| Person-years | 28450 | 185744 | 324665 | 149052 | 56624 |
| Incidence (events/100000 person-years) | 650 | 718 | 768 | 934 | 1174 |
| Adjusted HR (95% CI) | 0.81 (0.70-0.94) | 0.91 (0.86-0.98) | Ref. | 1.21 (1.13-1.29) | 1.46 (1.34-1.59) |
| **60-69 years** |  |  |  |  |  |
| Events | 296 | 2191 | 4119 | 2546 | 1463 |
| Person-years | 24833 | 163008 | 283876 | 159189 | 74952 |
| Incidence (events/100000 person-years) | 1192 | 1344 | 1451 | 1599 | 1952 |
| Adjusted HR (95% CI) | 0.79 (0.70-0.89) | 0.92 (0.87-0.97) | Ref. | 1.09 (1.04-1.15) | 1.29 (1.22-1.37) |
| **≥70 years** |  |  |  |  |  |
| Events | 191 | 1292 | 2316 | 1592 | 1000 |
| Person-years | 10333 | 61409 | 104797 | 66235 | 40861 |
| Incidence (events/100000 person-years) | 1848 | 2104 | 2210 | 2404 | 2447 |
| Adjusted HR (95% CI) | 0.82 (0.71-0.96) | 0.95 (0.88-1.01) | Ref. | 1.10 (1.03-1.17) | 1.11 (1.03-1.19) |

BP, blood pressure; HR, hazard ratio; CI, confidence interval.

**Supplementary Table S9. All-cause mortality of study subjects with anti-hypertensive medications according to BP and age categories**

| **Clinical event** | **<120/<70 mmHg** | **120-129/70-79 mmHg** | **130-139/80-89 mmHg** | **140-149/90-99 mmHg** | **≥150/≥100 mmHg** |
| --- | --- | --- | --- | --- | --- |
| **Total** |  |  |  |  |  |
| Events | 465 | 2541 | 4726 | 3194 | 2147 |
| Person-years | 25432 | 201899 | 403449 | 216629 | 100635 |
| Incidence (events/100000 person-years) | 1828 | 1259 | 1171 | 1474 | 2133 |
| Adjusted HR (95% CI) | 1.33 (1.21-1.46) | 1.01 (0.96-1.06) | Ref. | 1.15 (1.10-1.20) | 1.38 (1.31-1.46) |
| **<50 years** |  |  |  |  |  |
| Events | 15 | 105 | 219 | 162 | 77 |
| Person-years | 4025 | 36103 | 83255 | 39195 | 14002 |
| Incidence (events/100000 person-years) | 373 | 291 | 263 | 413 | 550 |
| Adjusted HR (95% CI) | 1.60 (0.94-2.72) | 1.12 (0.89-1.42) | Ref. | 1.51 (1.23-1.85) | 1.92 (1.47-2.49) |
| **50-59 years** |  |  |  |  |  |
| Events | 57 | 313 | 694 | 404 | 245 |
| Person-years | 8012 | 65262 | 132604 | 65691 | 26472 |
| Incidence (events/100000 person-years) | 711 | 480 | 523 | 615 | 926 |
| Adjusted HR (95% CI) | 1.51 (1.15-1.98) | 0.94 (0.82-1.08) | Ref. | 1.14 (1.01-1.29) | 1.63 (1.41-1.89) |
| **60-69 years** |  |  |  |  |  |
| Events | 179 | 957 | 1801 | 1174 | 806 |
| Person-years | 8915 | 71683 | 134233 | 78138 | 39441 |
| Incidence (events/100000 person-years) | 2008 | 1335 | 1342 | 1502 | 2044 |
| Adjusted HR (95% CI) | 1.48 (1.27-1.72) | 1.00 (0.93-1.08) | Ref. | 1.09 (1.01-1.18) | 1.45 (1.34-1.58) |
| **≥70 years** |  |  |  |  |  |
| Events | 214 | 1166 | 2012 | 1454 | 1019 |
| Person-years | 4478 | 28850 | 53355 | 33604 | 20718 |
| Incidence (events/100000 person-years) | 4779 | 4042 | 3771 | 4327 | 4918 |
| Adjusted HR (95% CI) | 1.23 (1.07-1.42) | 1.06 (0.99-1.14) | Ref. | 1.13 (1.06-1.21) | 1.22 (1.13-1.31) |

BP, blood pressure; HR, hazard ratio; CI, confidence interval.

**Supplementary Table S10. Cardiovascular mortality of study subjects with anti-hypertensive medications according to BP and age categories**

| **Clinical event** | **<120/<70 mmHg** | **120-129/70-79 mmHg** | **130-139/80-89 mmHg** | **140-149/90-99 mmHg** | **≥150/≥100 mmHg** |
| --- | --- | --- | --- | --- | --- |
| **Total** |  |  |  |  |  |
| Events | 138 | 656 | 1081 | 811 | 573 |
| Person-years | 25432 | 201899 | 403449 | 216629 | 100635 |
| Incidence (events/100000 person-years) | 543 | 325 | 268 | 374 | 569 |
| Adjusted HR (95% CI) | 1.70 (1.42-2.03) | 1.14 (1.03-1.25) | Ref. | 1.27 (1.16-1.40) | 1.61 (1.45-1.78) |
| **<50 years** |  |  |  |  |  |
| Events | 5 | 26 | 51 | 38 | 21 |
| Person-years | 4025 | 36103 | 83255 | 39195 | 14002 |
| Incidence (events/100000 person-years) | 124 | 72 | 61 | 97 | 150 |
| Adjusted HR (95% CI) | 2.62 (1.03-6.67) | 1.24 (0.77-2.00) | Ref. | 1.56 (1.02-2.37) | 2.34 (1.40-3.92) |
| **50-59 years** |  |  |  |  |  |
| Events | 12 | 73 | 133 | 85 | 56 |
| Person-years | 8012 | 65262 | 132604 | 65691 | 26472 |
| Incidence (events/100000 person-years) | 150 | 112 | 100 | 129 | 212 |
| Adjusted HR (95% CI) | 1.62 (0.89-2.94) | 1.14 (0.85-1.51) | Ref. | 1.28 (0.97-1.68) | 2.01 (1.47-2.75) |
| **60-69 years** |  |  |  |  |  |
| Events | 50 | 250 | 375 | 314 | 213 |
| Person-years | 8915 | 71683 | 134233 | 78138 | 39441 |
| Incidence (events/100000 person-years) | 561 | 349 | 279 | 402 | 540 |
| Adjusted HR (95% CI) | 1.96 (1.46-2.64) | 1.25 (1.07-1.47) | Ref. | 1.41 (1.21-1.64) | 1.86 (1.57-2.20) |
| **≥70 years** |  |  |  |  |  |
| Events | 71 | 307 | 522 | 374 | 283 |
| Person-years | 4478 | 28850 | 53355 | 33604 | 20718 |
| Incidence (events/100000 person-years) | 1586 | 1064 | 978 | 1113 | 1366 |
| Adjusted HR (95% CI) | 1.56 (1.22-2.00) | 1.07 (0.93-1.24) | Ref. | 1.13 (0.99-1.29) | 1.31 (1.13-1.52) |

BP, blood pressure; HR, hazard ratio; CI, confidence interval.

**Supplementary Table S11. Incidence of myocardial infarction of study subjects with anti-hypertensive medications according to BP and age categories**

| **Clinical event** | **<120/<70 mmHg** | **120-129/70-79 mmHg** | **130-139/80-89 mmHg** | **140-149/90-99 mmHg** | **≥150/≥100 mmHg** |
| --- | --- | --- | --- | --- | --- |
| **Total** |  |  |  |  |  |
| Events | 69 | 565 | 1019 | 571 | 351 |
| Person-years | 27079 | 208904 | 416419 | 226998 | 108596 |
| Incidence (events/100000 person-years) | 255 | 270 | 245 | 252 | 323 |
| Adjusted HR (95% CI) | 0.93 (0.73-1.19) | 1.06 (0.96-1.18) | Ref. | 1.00 (0.91-1.11) | 1.21 (1.07-1.37) |
| **<50 years** |  |  |  |  |  |
| Events | 3 | 71 | 138 | 70 | 19 |
| Person-years | 4089 | 36086 | 83412 | 39590 | 14355 |
| Incidence (events/100000 person-years) | 73 | 197 | 165 | 177 | 132 |
| Adjusted HR (95% CI) | 0.51 (0.16-1.60) | 1.22 (0.92-1.63) | Ref. | 1.05 (0.78-1.40) | 0.80 (0.49-1.29) |
| **50-59 years** |  |  |  |  |  |
| Events | 17 | 120 | 279 | 140 | 65 |
| Person-years | 8169 | 65893 | 133840 | 66785 | 27343 |
| Incidence (events/100000 person-years) | 208 | 182 | 208 | 210 | 238 |
| Adjusted HR (95% CI) | 1.06 (0.64-1.73) | 0.89 (0.71-1.10) | Ref. | 1.02 (0.83-1.25) | 1.14 (0.87-1.50) |
| **60-69 years** |  |  |  |  |  |
| Events | 22 | 258 | 381 | 229 | 156 |
| Person-years | 9495 | 73838 | 139153 | 81620 | 42209 |
| Incidence (events/100000 person-years) | 232 | 349 | 274 | 281 | 370 |
| Adjusted HR (95% CI) | 0.80(0.52-1.23) | 1.26(1.08-1.48) | Ref. | 1.02(0.87-1.20) | 1.33 (1.10-1.61) |
| **≥70 years** |  |  |  |  |  |
| Events | 27 | 116 | 221 | 132 | 111 |
| Person-years | 5324 | 33085 | 60013 | 39001 | 24688 |
| Incidence (events/100000 person-years) | 507 | 351 | 368 | 338 | 450 |
| Adjusted HR (95% CI) | 1.30 (0.87-1.95) | 0.94 (0.75-1.17) | Ref. | 0.92 (0.74-1.14) | 1.19 (0.95-1.50) |

BP, blood pressure; HR, hazard ratio; CI, confidence interval.

**Supplementary Table S12. Incidence of stroke of study subjects with anti-hypertensive medications according to BP and age categories**

| **Clinical event** | **<120/<70 mmHg** | **120-129/70-79 mmHg** | **130-139/80-89 mmHg** | **140-149/90-99 mmHg** | **≥150/≥100 mmHg** |
| --- | --- | --- | --- | --- | --- |
| **Total** |  |  |  |  |  |
| Events | 241 | 2014 | 4108 | 2598 | 1548 |
| Person-years | 26161 | 200354 | 397456 | 214220 | 101031 |
| Incidence (events/100000 person-years) | 921 | 1005 | 1034 | 1213 | 1532 |
| Adjusted HR (95% CI) | 0.78 (0.69-0.89) | 0.92 (0.88-0.97) | Ref. | 1.11 (1.06-1.17) | 1.26 (1.19-1.34) |
| **<50 years** |  |  |  |  |  |
| Events | 14 | 126 | 345 | 184 | 104 |
| Person-years | 4031 | 35821 | 82002 | 38877 | 13855 |
| Incidence (events/100000 person-years) | 347 | 352 | 421 | 473 | 751 |
| Adjusted HR (95% CI) | 0.84 (0.49-1.44) | 0.83 (0.68-1.02) | Ref. | 1.13 (0.95-1.35) | 1.80 (1.44-2.25) |
| **50-59 years** |  |  |  |  |  |
| Events | 52 | 430 | 941 | 566 | 258 |
| Person-years | 7977 | 64011 | 129586 | 64045 | 26071 |
| Incidence (events/100000 person-years) | 652 | 672 | 726 | 884 | 990 |
| Adjusted HR (95% CI) | 0.86 (0.65-1.13) | 0.90 (0.81-1.01) | Ref. | 1.21 (1.09-1.35) | 1.30 (1.13-1.49) |
| **60-69 years** |  |  |  |  |  |
| Events | 97 | 881 | 1709 | 1100 | 685 |
| Person-years | 9114 | 70183 | 130981 | 76277 | 38891 |
| Incidence (events/100000 person-years) | 1064 | 1255 | 1305 | 1442 | 1761 |
| Adjusted HR (95% CI) | 0.79 (0.64-0.97) | 0.96 (0.88-1.04) | Ref. | 1.09 (1.01-1.18) | 1.30 (1.19-1.42) |
| **≥70 years** |  |  |  |  |  |
| Events | 78 | 577 | 1113 | 748 | 501 |
| Person-years | 5038 | 30337 | 54886 | 35019 | 22212 |
| Incidence (events/100000 person-years) | 1548 | 1902 | 2028 | 2136 | 2256 |
| Adjusted HR (95% CI) | 0.75 (0.60-0.95) | 0.93 (0.84-1.02) | Ref. | 1.06 (0.96-1.16) | 1.10 (0.99-1.23) |

BP, blood pressure; HR, hazard ratio; CI, confidence interval.
